# Rhythmic arm swing enhances patterned locomotor-like muscle activity in passively moved lower extremities

**DOI:** 10.14814/phy2.12317

**Published:** 2015-03-05

**Authors:** Tetsuya Ogawa, Takahiko Sato, Toru Ogata, Shin-Ichiro Yamamoto, Kimitaka Nakazawa, Noritaka Kawashima

**Affiliations:** 1 Faculty of Sport Sciences, Waseda UniversityTokorozawa, Saitama, Japan; 2 Japan Society for the Promotion of ScienceChiyoda, Tokyo, Japan; 3 Department of Rehabilitation for the Movement Functions, Research Institute, National Rehabilitation Center for Persons with DisabilitiesTokorozawa, Japan; 4 College of Systems Engineering and Science, Shibaura Institute of TechnologyMinuma, Saitama, Japan; 5 Graduate School of Arts and Sciences, The University of TokyoMeguro, Tokyo, Japan

**Keywords:** Arm swing, locomotor-like EMG, Lokomat, passive stepping

## Abstract

The use of driven gait orthosis (DGO) has drawn attention in gait rehabilitation for patients after central nervous system (CNS) lesions. By imposing a passive locomotor-like kinematic pattern, the neural mechanisms responsible for locomotion can be activated as in a normal gait. To further enhance this activity, discussions on possible intervention are necessary. Given the possible functional linkages between the upper and lower limbs, we investigated in healthy subjects the degree of modification in the lower limb muscles during DGO-induced passive gait by the addition of swing movement in the upper extremity. The results clearly showed that muscle activity in the ankle dorsiflexor TA muscle was significantly enhanced when the passive locomotor-like movement was accompanied by arm swing movement. The modifications in the TA activity were not a general increase through the stride cycles, but were observed under particular phases as in normal gaits. Voluntary effort to swing the arms may have certain effects on the modification of the muscle activity. The results provide clinical implications regarding the usefulness of voluntary arm swing movement as a possible intervention in passive gait training using DGO, since ordinary gait training using DGO does not induce spontaneous arm swing movement despite its known influence on the lower limb movement.

## Introduction

Over the last decade, the use of driven gait orthosis (DGO) has been considered in gait rehabilitation after central nervous system (CNS) lesion, especially in spinal cord-injured (SCI) patients (Colombo et al. 2001; Hornby et al. 2005; Wirz et al. 2005) and poststroke patients (Husemann et al. 2007; Westlake and Patten 2009). By imposing locomotor-like passive movement in the lower extremities, neural mechanisms responsible for generating gait movement can be activated, as demonstrated in the phase-dependent modulation of both the monosynaptic H-reflex (Kamibayashi et al. 2010) and the cutaneous reflex (Nakajima et al. 2008a,b) in specific lower limb muscles.

Recent studies have revealed possible interactions between the upper and lower extremities. For example, Nakajima et al. (2011) found that H-reflex responses in a stationary-fixed forearm flexor carpi radialis (FCR) muscle were remotely modulated during passive leg movement induced by DGO. Conversely, Kawashima et al. (2008) showed in cervical incomplete SCI patients that the addition of passive arm movement during alternate locomotor-like movement in the lower extremities modulated activities in lower leg muscles under certain phases. In particular, they demonstrated that activities in the plantarflexor soleus (SOL) muscle were both enhanced and suppressed in a phase-dependent manner, resembling features of physiological gait (Kawashima et al. 2008). More direct evidence demonstrated the possible involvement of particular neural linkages between the upper and lower extremities. When subjects engaged in cycling movement with their upper extremities, the amplitudes of the H-reflex and the stretch reflex in the lower extremity SOL muscles were significantly suppressed under specified phases of arm cycling compared to those under identical arm positions but kept stationary (Frigon et al. 2004; Loadman and Zehr 2007; Palomino et al. 2011).

With the use of DGO, the passive lower limb movement does not induce apparent swinging behavior in the upper limb. However, given the possible functional linkage between the upper and lower extremities (Frigon et al. 2004; Loadman and Zehr 2007; Kawashima et al. 2008; Nakajima et al. 2011; Palomino et al. 2011), the addition of any upper limb movement may have certain effects on the activities of the lower limb muscles.

The purpose of this study was to investigate the effects of arm swing movement on the activity of muscles in a lower extremity that is passively moved in a locomotor-like manner using DGO. In light of the results of preceding studies, we hypothesized that arm-swing movements during passive stepping amplifies the phase-dependent modulation of the muscle activities in the lower extremity (i.e., both enhanced and weakened in a phase-specific manner). The possible changes in the muscle activities with this intervention may provide insights into a construction of rehabilitation protocols using DGO for patients after CNS lesion.

## Methods

### Participants

The participants were 10 healthy male volunteers with no known history of neurological or orthopedic disorders (mean age: 24.2 ± 5.8 years). They provided informed consent for the experimental procedures prior to participation. This study was approved by the local ethics committee of the National Rehabilitation Center for Persons with Disabilities, Japan and was conducted in accordance with the Declaration of Helsinki.

### Stepping movement

**Figure 1:**
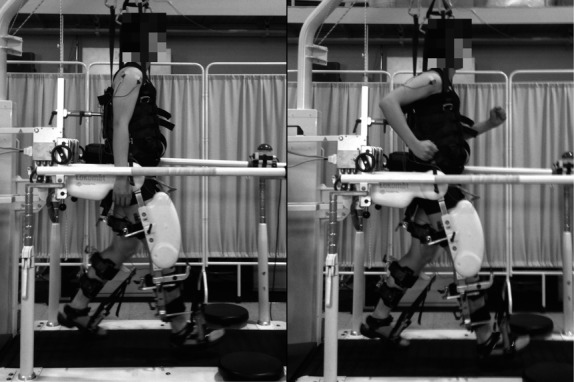
The experimental apparatus and the tasks used. Each subject walked passively in the DGO and kept his lower extremities relaxed throughout the session, with different tasks for the upper extremities. In the first experiment, the subject walked without (left panel) and with (right panel) arm swing. In the second experiment, he walked with the arms swinging while wearing different loads on both wrists.

In this study, subjects walked passively in a DGO apparatus (Lokomat®, Hocoma, Volketswil, Switzerland; Fig.1) with several different movement conditions for the upper extremities. The details of the DGO are described elsewhere (Colombo et al. 2001). Briefly, by actuators mounted at the joints of the orthosis, the Lokomat provides the subject with hip and knee joint movements that resemble normal walking on a treadmill. The movement of the ankle joint is supported toward dorsiflexion by a passive foot-lifter during the swing phase; during the stance phase, the movement of the treadmill controls the movement of the feet (Colombo et al. 2001). Upon setup, the DGO was secured to the subject with two straps; one around the pelvis and another around the chest. The subject's lower limb segments were then fixed to the respective segments of the orthosis, which were preliminarily adjusted to the subject for each segment length. For smooth movement of the device, the center of rotation of each joint was precisely adjusted between the subjects and the orthosis in the sagittal plane. Lastly, the passive foot-lifter (a vinyl strap and spring attached at the front of the foot) was fixed with the ankle angle at 90° while the subject stood upright. With the foot-lifter, dorsiflexion during the swing phase was achieved and therefore, appropriate clearance could be maintained without any activation of the dorsiflexor muscles.

### Experimental protocols

Before the recording was conducted, the subjects were given a familiarization session lasting for approx. 3 min. During this session, the subject learned to walk “passively”; that is, to completely relax and be moved by the orthosis. Also in this session, adjustment of the passive foot-lifter was done to ensure that the subject could maintain constant clearance during the swing phase. The treadmill speed was maintained at 3.0 km h^−1^ throughout the entire experimental session, and the body weight of each subject was unloaded by approx. 85% by a body weight-unloading system mounted on the device. The use of the body weight unloading with this amount was based on our preceding results showing that locomotion-related neural responses during a passive stepping task took place under the loaded condition regardless of the amount of loading (Nakajima et al. 2008b).

With the lower extremities constantly relaxed while walking passively, the subjects were first given two tasks: either (1) with or (2) without arm swinging to investigate the influence of arm swing on the activities of the lower limb muscles. They subsequently went through four different arm swinging tasks with the addition of weights (0, 0.75, 1.5, and 2.25 kg) around both wrists. These additional weights were utilized to modify the extent of voluntary effort to swing the arms. The order of exposure to the different task conditions was randomized across subjects. The addition of weights around the wrists was compensated for by the addition of body weight unloading to maintain a constant load acting on the soles of the feet. During the no-arm swing session, the subject stayed relaxed with the hands on the side of the orthosis. During the arm swing sessions, the subject was instructed to swing his arms spontaneously with the elbows bent at approx. 90°. This was necessary to allow natural forward–backward arm swinging in the sagittal plane because of the existence of the orthosis on the subject's sides encumbering natural arm swing (with nearly straightened elbows) as in a normal gait.

To maintain a constant range of motion (ROM) during the arm swing, the shoulder angles in the sagittal plane (i.e., flexion and extension) on both sides were visually fed back to the subject online with an oscilloscope placed 2 m in front of the subject. Target signals were provided on the basis of the natural behavior of each subject during the familiarization session. The recordings under each task condition lasted 90 sec.

### Recordings

Electromyographic (EMG) activity in the tibialis anterior (TA), the medial head of the gastrocnemius (MG), the rectus femoris (RF), the biceps femoris (BF), the anterior head of deltoid (aDEL), and the posterior head of deltoid (pDEL) muscles on the right side was recorded using bipolar electrodes. For the muscles in the lower extremities, the electrodes were placed by avoiding the cuffs of the orthosis. Prior to the placement of the electrodes, the surface of the skin at the site of the placement was rubbed lightly with sandpaper and cleaned with alcohol pads. The EMG signals obtained were amplified (Bagnoli-8 EMG system, Delsys, Boston, MA) and band-pass filtered (between 20 and 450 Hz). The angles of the ankle and shoulder joints in the sagittal plane were measured bilaterally by goniometers attached across each joint. The hip and knee joint angles were measured by potentiometers mounted in the orthosis. The EMG signals along with the joint angles were sampled at 1 kHz using an A/D converter (PowerLab, AD Instruments, Colorado Springs, CO) and stored in a computer for later analysis. The load acting on the foot soles during the passive walking was sampled from five of the subjects using the F-scan system (Nitta Corp., Tokyo, Japan) at 30 Hz.

### Data analysis and statistics

In each 90-sec recording session under respective tasks, the data during the last 30 sec were analyzed in order to eliminate the history of the preceding tasks. The EMG signals were full-wave rectified after subtraction of the direct current (DC) component. The EMG signals, along with the joint angles, were then binned into stride cycles on the basis of maximal hip extension, and they were ensemble averaged over the stride cycles for each subject (approx. 40 stride cycles per task). We calculated the EMG amplitude and the joint angles from the ensemble-averaged values. The EMG amplitude was measured as an integrated value over the stride cycle. Load acting on the foot soles was calculated on the basis of the net force during the whole stance phase.

Statistical analyses were performed with factors of muscles activities, the ROM in the joint angles, and the load acting on the foot soles among the different tasks with the upper extremities. First, to address the changes in these variables that depend solely on the presence or absence of the arm swing, Student's *t*-tests for paired samples were performed for each variable between with and without arm swing. To test for the influence of the additional load at the wrists on the muscle activities, a one-way ANOVA with repeated measures (four weights) was used. To address the changes in testing conditions, a one-way ANOVA with repeated measures was performed for joint ROM and the load acting on the foot soles among the conditions with different loading at the wrist. Student's *t*-tests for paired samples were performed for joint ROM and load acting on foot soles between the arm swing and no-arm swing conditions. For ANOVA comparisons, when the result of Mauchly's sphericity test was less than 0.05, we used corrected values obtained by Greenhouse-Geisser correction. When there were significant main effects, Bonferroni's post-hoc comparisons were performed. Correlation coefficients were calculated to test for the relationships among variables. Data are presented as the mean values and standard deviation (mean ± SD). Significance was accepted at *P* < 0.05.

## Results

**Figure 2:**
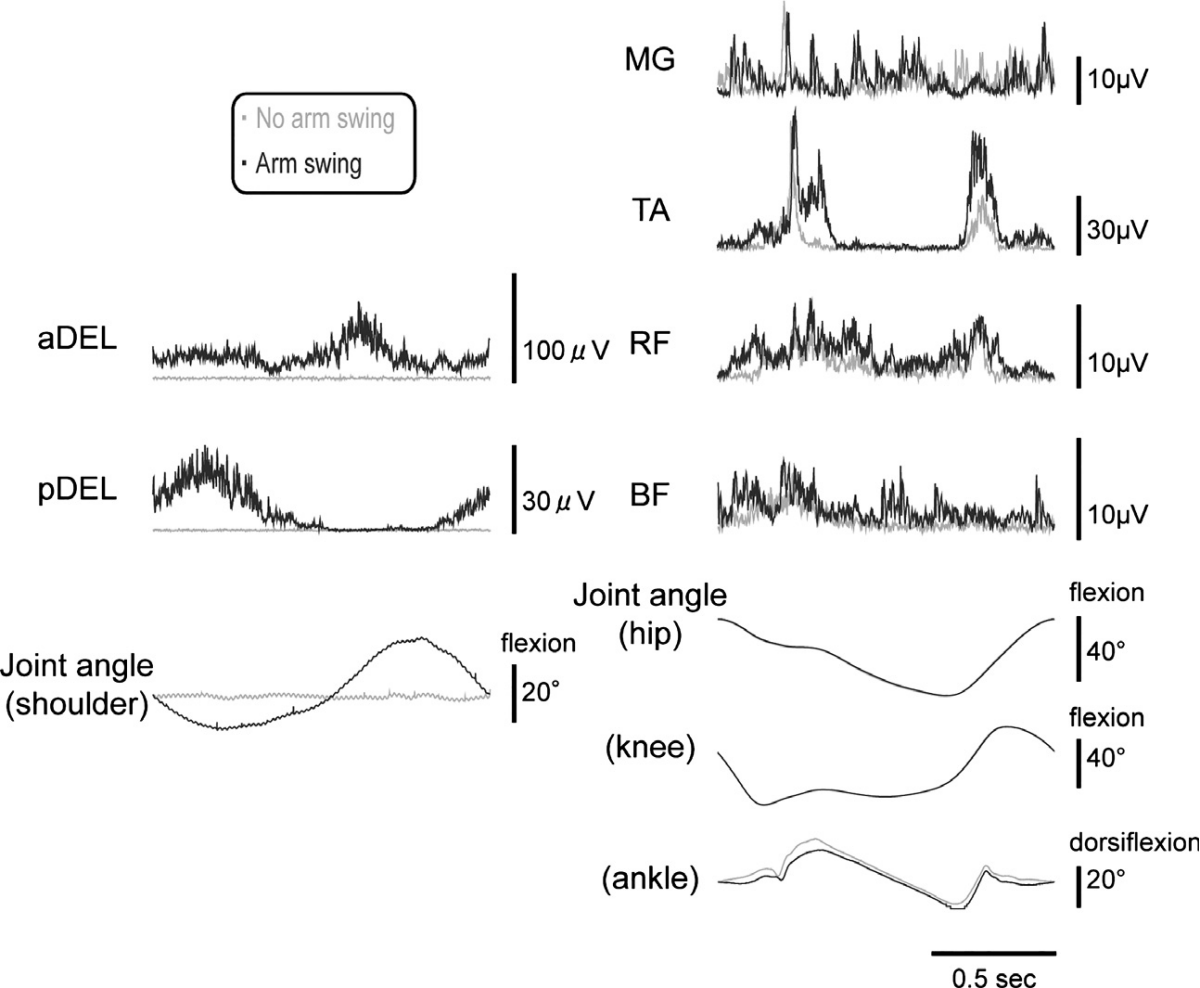
Representative EMG waveforms for the muscles investigated and the joint movements during gait cycles in both the upper and lower extremities. Each waveform represents the ensemble average of gait cycles over 30 sec. Gray waveforms are those without arm swing; black lines are those with arm swing.

The statistical analysis revealed no significant differences in the load acting on the foot soles between the tasks with and without arm swinging (*P* = 0.786), or among the conditions with different additional loading at the wrist (*F* = 1.284, df = 1.244, *P* = 0.325). There were no significant differences in the ROM in the passively walking hip (*P* = 0.229) (*F* = 0.608, df = 1.834, *P* = 0.543), knee (*P* = 0.261) (*F* = 0.616, df = 3, *P* = 0.610), and ankle (*P* = 0.856) (*F* = 0.398, df = 1.896, *P* = 0.667) joints both between and among the above-mentioned tasks (representative waveforms are shown in Figs2 and 4). The ROM in the shoulder also showed no significant differences (*F* = 0.677, df = 1.663, *P* = 0.497) among the different additional wrist loads.

Figure2 portrays representative waveforms of the EMG activities and the joint angles with the presence (black lines) or absence (gray lines) of arm swing. Each waveform represents an ensemble average of stride cycles over 30 sec for analysis (approx. 40 stride cycles) from a single subject. In contrast to the upper extremity muscles where the activities were silent during the no-arm swinging session, the lower limb muscles already showed some phasic bursts although the subject attempted to keep relaxed.

**Figure 3:**
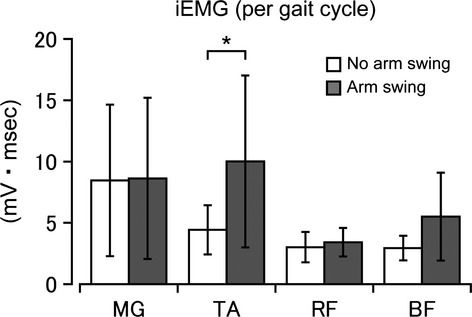
Comparisons of mean EMG values in each muscle investigated between the with/without arm swing conditions. Error bars: standard deviations (SD). Statistically significant difference *P < 0.05.

The addition of arm swinging resulted in clear phase-dependent EMG bursts in the upper extremity muscles. Furthermore, the activities in some muscles in the lower extremities were amplified by the addition of arm swinging even though the lower extremities were only “passively” walking. Figure3 compares The activities in the lower leg muscles between the no-arm swing and the arm swing conditions in the four lower limb muscles tested. There was significant difference between the arm swing and no-arm swing conditions in the TA muscle (*P* < 0.05). The other muscles did not show any significant differences (MG: *P* = 0.909; RF: *P* = 0.141; BF: *P* = 0.076).

**Figure 4:**
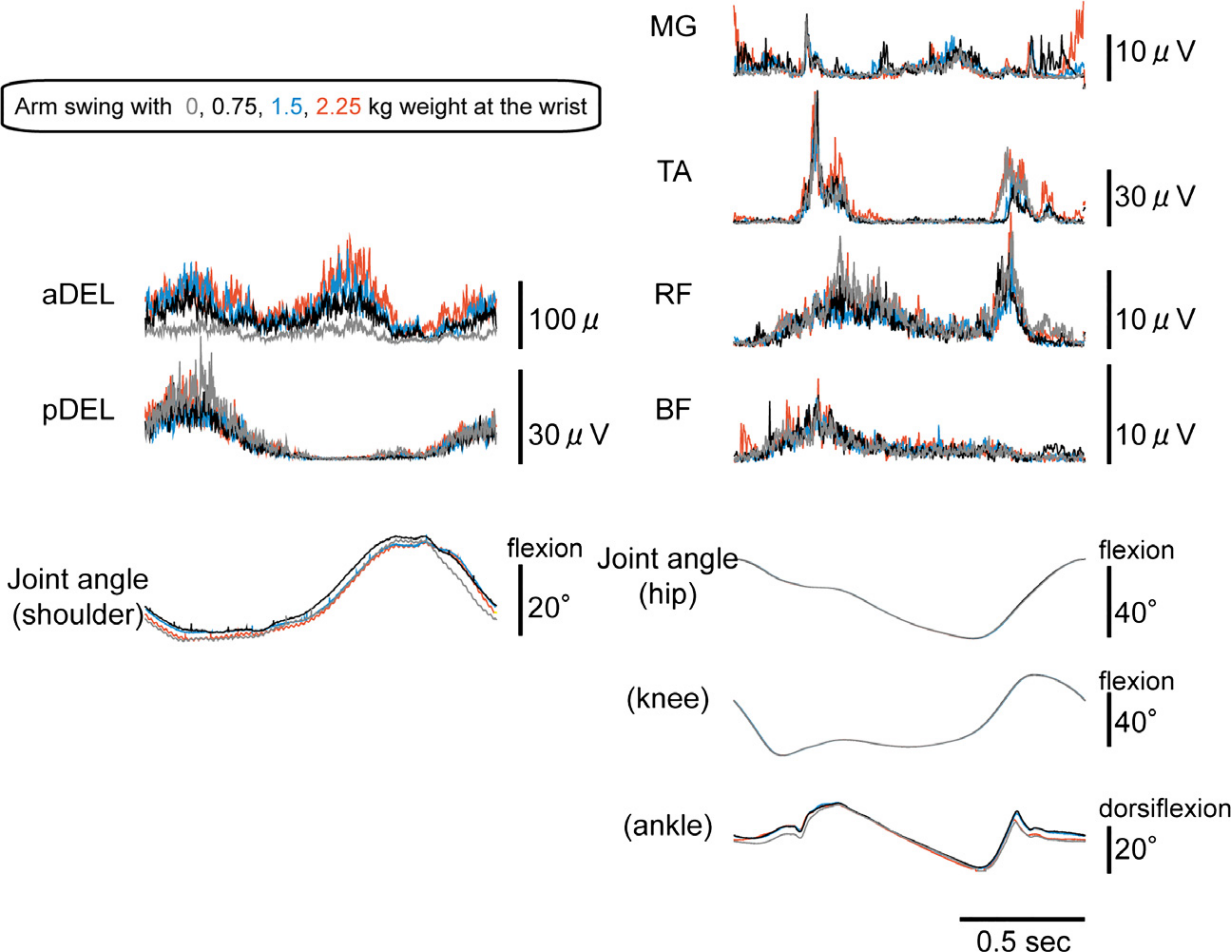
Representative EMG waveforms for the muscles investigated and the joint movements during gait cycles in both the upper and lower extremities under different weight loads (0, 0.75, 1.5, and 2.25 kg) applied to the wrists of the swinging arms. Each waveform represents the ensemble average of gait cycles over 30 sec.

**Figure 5:**
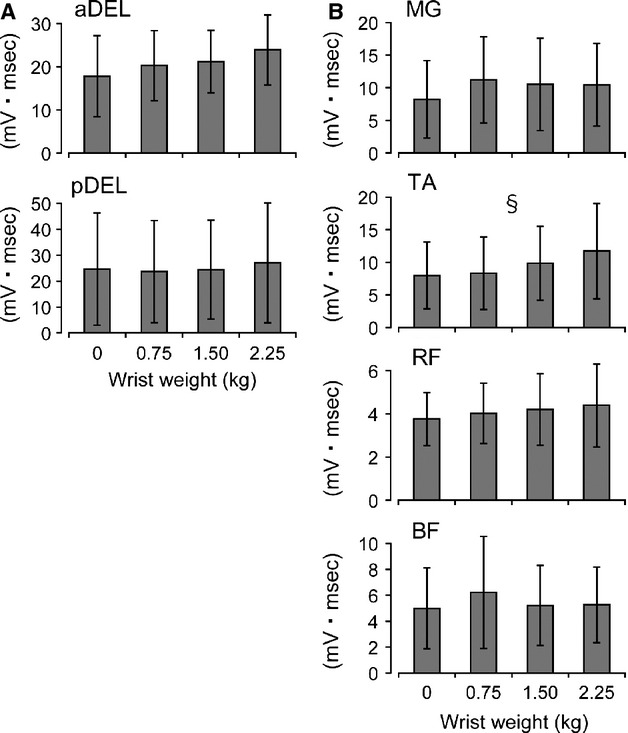
Mean EMG values in each muscle investigated among different conditions of arm swinging (accompanying different weight loads at the wrist). Error bars: SD. Statistically significant main effect §*P* < 0.05

A representative example of how arm swing with the addition of wrist weights modulated activity in the respective muscles is shown in Figure4. In the upper extremities, the activity in the aDEL tended to be modulated in a weight-dependent manner, whereas the pDEL remained constant despite the addition of weight. In the lower extremity muscles, some aspect of modulation could be seen depending on the muscle (most prominent in the TA muscle). In the muscles tested, the statistical analysis showed a significant main effect in the TA muscle with changing wrist weight (*F* = 2.997, df = 3, *P* < 0.05) (Fig.5). Post-hoc comparisons did not reveal any significant differences between variables. Among the other muscles, the upper extremity aDEL muscle tended to be modulated with changing wrist weight (*F* = 2.806, df = 3, *P* = 0.059), but not significantly so, along with the other muscles tested (MG: *F* = 2.168, fd = 1.549, *P* = 0.158; RF: *F* = 1.466, fd = 1.762, *P* = 0.259; BF: *F* = 0.546, fd = 1.901, *P* = 0.580; pDEL: *F* = 2.725, df = 1.673, *P* = 0.105).

**Figure 6:**
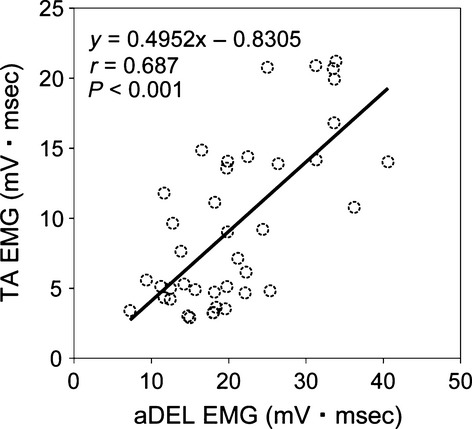
Relationship between muscle activity in the upper extremity (aDEL) and the ankle dorsiflexor TA muscle under arm swing with different weights applied at the wrist (0, 0.75, 1.5, and 2.25 kg) in the 10 subjects. There was statistically significant correlation with *P* < 0.001.

The relationship between muscle activity in the upper extremity aDEL and the ankle dorsiflexor TA muscle under the arm swing condition with different weight at the wrist (0, 0.75, 1.5, and 2.25 kg) is shown in Figure6. There was a significant correlation between the variables (*P* < 0.001). In contrast, there were no correlations between activity in the pDEL and the TA muscles (data not shown).

## Discussion

We investigated the effects of voluntary arm swing movement on the activity of muscles in the lower extremities moved passively by DGO in a locomotor-like kinematic pattern. The results show that (1) the activity in the dorsiflexor TA muscle was significantly larger when the passive leg movement was accompanied by arm swinging compared to that under the no-arm swinging condition, and (2) the activity in the TA muscle was further amplified by the addition of a weight load at the wrists of the swinging arms. The modulation was identified in the TA muscle and not in any of the other muscles investigated.

### Neural control of normal gait and arm swinging

It is well accepted that the motor activities found in human locomotion are not solely a consequence of voluntary effort or the simple sum of reflex responses induced by associated joint movements (Dietz 2002). Instead, a network of interneurons in the spinal cord, called the central pattern (or rhythm) generator (CPG) (Calancie et al. 1994; Dietz 2002) is known to exist and to play a significant role in the generation of muscle activities. Based on the phase-dependent contribution of corticospinal input (Schubert et al. 1997; Kamibayashi et al. 2009) and afferent input from the periphery (Duysens and Van de Crommert 1998; Kawashima et al. 2005), the CPG consequently provides the lower limb muscles with patterned neural commands that in turn result in joint movements. Even in passive gaits where lower limb movements are generated by external support using DGO, it has been demonstrated that corticospinal excitability (Kamibayashi et al. 2009) and reflex responses (Nakajima et al. 2008a,b; Kamibayashi et al. 2010) in lower limb muscles were modulated in a phase-dependent manner, indirectly showing the capability of the CPG.

Along with the lower limb movement, in human walking, we rhythmically and alternately swing our arms unconsciously. Several investigations have provided possible explanations of the functional significance of this arm swinging. For instance, Collins et al. (2009) demonstrated that disturbance in natural arm swing while walking resulted in excessive momentum in the arms and the whole body, and consequently the vertical ground reaction moment. Moreover, arm swing was shown to have a significant role in reducing moments around the vertical axes in the lower limbs (Park 2008) and angular momentum within the whole body (Herr and Popovic 2008). Together these results from the biomechanical perspective demonstrate the importance of the arm swing movement in bipedal human gait for the conservation of mechanical equilibrium.

In the emergence of this spontaneous behavior (i.e., arm swinging during normal gait), it is known that several muscles, especially those around the shoulder joint, are recruited in a phase-dependent manner (Kuhtz-Buschbeck and Jing 2012). This contribution of the muscles indicates that arm swing is not simply a passive behavior as a counter-movement to the motion of the trunk and lower extremities, but rather that it is more actively (indicating neurally) controlled (Kuhtz-Buschbeck and Jing 2012). As found in the lower limb movements, arm swing during walking has also been described as being under the control of the CPG (for a review, see Zehr and Duysens 2004). This notion of the CPG underlying arm swing movement has been demonstrated predominantly by phase-dependent modulation in the amplitude of the H-reflex (Zehr et al. 2003) and the phase-dependent modulation and reversal in cutaneous reflex responses (Zehr and Kido 2001) during arm-cycling movement.

### The significance of voluntary arm swing in this study

The voluntary arm swinging investigated here, then, may be in conflict with the nature of the physiological gait, in which the motor patterns – for the most part – are generated automatically. However, this study was based on the use of DGO with its expected use in gait rehabilitation in the future, in which arm swing movement was not induced spontaneously. Any intervention to facilitate locomotor activities in the lower limb muscles can play an important role in the training processes. Prior studies of the effects of voluntary upper limb movements on the magnitude of spinal reflex responses in the lower limb muscles (Frigon et al. 2004; Loadman and Zehr 2007; Palomino et al. 2011) have suggested the potential of voluntary arm swing as a possible intervention for improving lower limb muscle activities.

This study is the first to address the influence of voluntary arm swinging on lower limb muscle activities during passive gait using DGO. Several investigations demonstrated the effects of upper limb movement on the activity of lower limb muscles while the subject is seated at rest (Frigon et al. 2004; Loadman and Zehr 2007; Palomino et al. 2011) or during passive movement of the lower limb coupled to the upper limb while seated on a recumbent stepper (Huang and Ferris 2004). However, the results obtained under these postural conditions suggested a possible lack of afferent information including that from the hip joint and load receptors that constitute a fundamental part of the natural gait (Andersson and Grillner 1983; Kriellaars et al. 1994; Pearson 1995; Dietz and Duysens 2000; Dietz 2002). Hence, this study using DGO with the subjects standing upright provided a situation closer to the nature of normal gait.

### Modification of activities in lower limb muscles with voluntary arm swinging

The use of DGO allowed emergences of phasic EMG bursts in some lower limb muscles despite the passive movement with no voluntary effort by the subjects (as shown in the representative waveforms in Fig.2). With the addition of the arm swing movement, the EMG activities through the stride cycles were greater than those without the arm swing. The greater EMG activity took place only in the dorsiflexor TA muscle and not in the other muscles investigated. The increased activity in the TA muscle, as shown in Figure2, was not just a general increment through the stride cycles, but rather it was amplified under some phases and stayed silent under other phases. This is consistent with the results in a preceding study in which muscle activity in the lower extremity during passive locomotor-like movement was shaped rather than exhibiting a general increment or decrement through the movement cycle (Kawashima et al. 2008).

In this study, we did not record the moments of foot contact and toe-off, but in accord with results reported by Kamibayashi et al. (2010), the phases corresponded to the early stance phase near foot contact and the early swing phases, respectively. These phases are where the activity in the TA muscle is evident in normal gait as well (De Serres et al. 1995). Functionally, the TA muscle has a significant role in the control of the lowering of the foot after heel contact (i.e., the early stance phase) and in the clearing of the foot from the ground (the early swing phase) (De Serres et al. 1995).

Why then did the amplification of the muscle activity take place only in the TA and not in other muscles? With respect to the results in which the EMG responses were further enhanced by the addition of weight on the swinging arms (as demonstrated by the significant main effect for different weights), it is likely that the extent of voluntary effort responsible for the upper limb movement played a significant role. This is in accordance with the results reported by Hundza and Zehr (2009): using an arm-cycling movement, they showed that the degree of suppression in the soleus H-reflex while sitting at rest was modified under different movement frequencies of arm cycling (therefore, under different voluntary efforts to move the arms). Furthermore, the result where the muscle activity in the shoulder flexor aDEL was highly correlated with that in the ankle dorsiflexor TA muscle further supports the possible involvement of voluntary efforts. By addressing modifications in motor evoked potentials (MEPs) induced by transcranial magnetic stimulation (TMS) that coincided with stretch reflex responses, Christensen et al. (2001) demonstrated that corticospinal excitability to the TA muscle was enhanced in both the stance phase and the swing phase. Of particular interest, the enhancement took place when the TMS was timed to the appearance of the late component of the stretch reflex (Christensen et al. 2001), indicating a contribution of a transcortical reflex pathway.

On the other hand, it is known that unintended muscle activity can occur upon performing muscle contractions above certain contraction levels (so-called “neural cross-talk”). For instance, an attempt to generate muscle activity in a particular muscle resulted in the unintentional activation of other muscles in the same limb (Dimitrijevic et al. 1992) and in the contralateral limb (Dimitrijevic et al. 1992; Arányi and Rösler 2002). Collectively, the results of these studies and the present findings suggest that the enhanced activity in the TA muscle with the addition of arm swinging may be a consequence of increased corticospinal excitability under particular phases accompanied by an unintentional neural command to the responsible motor area for the lower limb movement induced by the upper limb movement. Regarding the contrasting results between the aDEL and the pDEL in the relationship with the TA muscle, we suspect that the functional differences in these muscles may provide a possible explanation. In rhythmic movements of two different limb segments (hand and foot), Baldissera et al. (1982, 1991) showed that there is a preference in the association of the joint movements. They observed that the movements were easily performed when the two limb segments were moved in-phase (into same directions). Given the function of the muscles tested here (the TA and aDEL as flexors and the pDEL as an extensor), the present finding of a significant correlation in the EMG activity between the aDEL and the TA and not between the pDEL and the TA may be due to the difference in the functional linkage between the respective combination of the muscles.

Another possibility could be based on the mechanisms inherent in the spinal cord connecting the cervical and lumber spinal cord. A population of neurons in the spinal cord, so-called propriospinal neurons, is thought to play a significant role in the coordination of neural functions between the forelimbs and hindlimbs of cats, as found in the coordination patterns in locomotive movement (Miller et al. 1975) and interlimb reflex responses (Miller et al. 1973). On the basis of the connections in the animal models, recent studies on humans have elucidated the contribution of the upper limb movements to the motor activities in the lower extremities (Frigon et al. 2004; Loadman and Zehr 2007; Kawashima et al. 2008; Palomino et al. 2011).

In contrast to the TA muscle with its enhancement by arm swinging, the reasons for the lesser modification in the other muscles remain unclear. One possibility is associated with the characteristic features in those muscles as extensors. Extensor muscles are known to be sensitive to excitatory input derived from the sensory input from the load receptors (Duysens and Pearson 1980). Their activities may thus be less influenced by the descending neural command from the supraspinal center that, in turn, results in only a minor modification by the addition of arm swing movement.

In this study, several limitations arise from the characteristics of the experimental apparatus. Because of the existence of the orthosis on both sides of the lower extremities, the subjects’ natural arm swing (with nearly straight elbows) as in normal walking was encumbered. The subjects were therefore instructed to swing their bent arms. This unnatural arm swing with shortened moment arm may have affected the muscle activity of the lower extremities differently from normal walking. Furthermore, in this study, the subjects’ natural pelvic movement was affected by the fixation to the orthosis. In light of the report by Kamibayashi et al. (2010) showing the possible contribution of movement-related joint afferents from the lower limb to the locomotion-related neural function, we suspect that the use of an apparatus with an increased degree of freedom in the pelvic movement may provide results closer to those of natural walking. Despite these limitations, this study can be regarded as the first step to consider possible interventions for gait rehabilitation using DGO.

To summarize, the addition of arm swing movement to passive stepping movement in the lower limbs resulted in a clear amplification of activities in the dorsiflexor TA muscle. Our results provide insight into the use of this paradigm in the field of gait rehabilitation. Further investigations of the detailed neural mechanisms and techniques to activate other muscle groups (e.g., by appropriately modifying the weight load as the occasion demands) are needed before any clinical applications are considered, however.

## Conflict of Interest

The authors have declared that no competing interests exist.
